# Computational Investigations of Learning and Synchronization in Cognitive Control

**DOI:** 10.5334/joc.239

**Published:** 2022-09-30

**Authors:** Pieter Huycke, Elise Lesage, C. Nico Boehler, Tom Verguts

**Affiliations:** 1 Department of Experimental Psychology, Ghent University, BE

**Keywords:** Learning, synchronization, binding, cognitive control

## Abstract

Complex cognition requires binding together of stimulus, action, and other features, across different time scales. Several implementations of such binding have been proposed in the literature, most prominently synaptic binding (learning) and synchronization. Biologically plausible accounts of how these different types of binding interact in the human brain are still lacking. To this end, we adopt a computational approach to investigate the impact of learning and synchronization on both behavioral (reaction time, error rate) and neural (θ power) measures. We train four models varying in their ability to learn and synchronize for an extended period of time on three seminal action control paradigms varying in difficulty. Learning, but not synchronization, proved essential for behavioral improvement. Synchronization however boosts performance of difficult tasks, avoiding the computational pitfalls of catastrophic interference. At the neural level, θ power decreases with practice but increases with task difficulty. Our simulation results bring new insights in how different types of binding interact in different types of tasks, and how this is translated in both behavioral and neural metrics.

## Introduction

**Figure 1:**
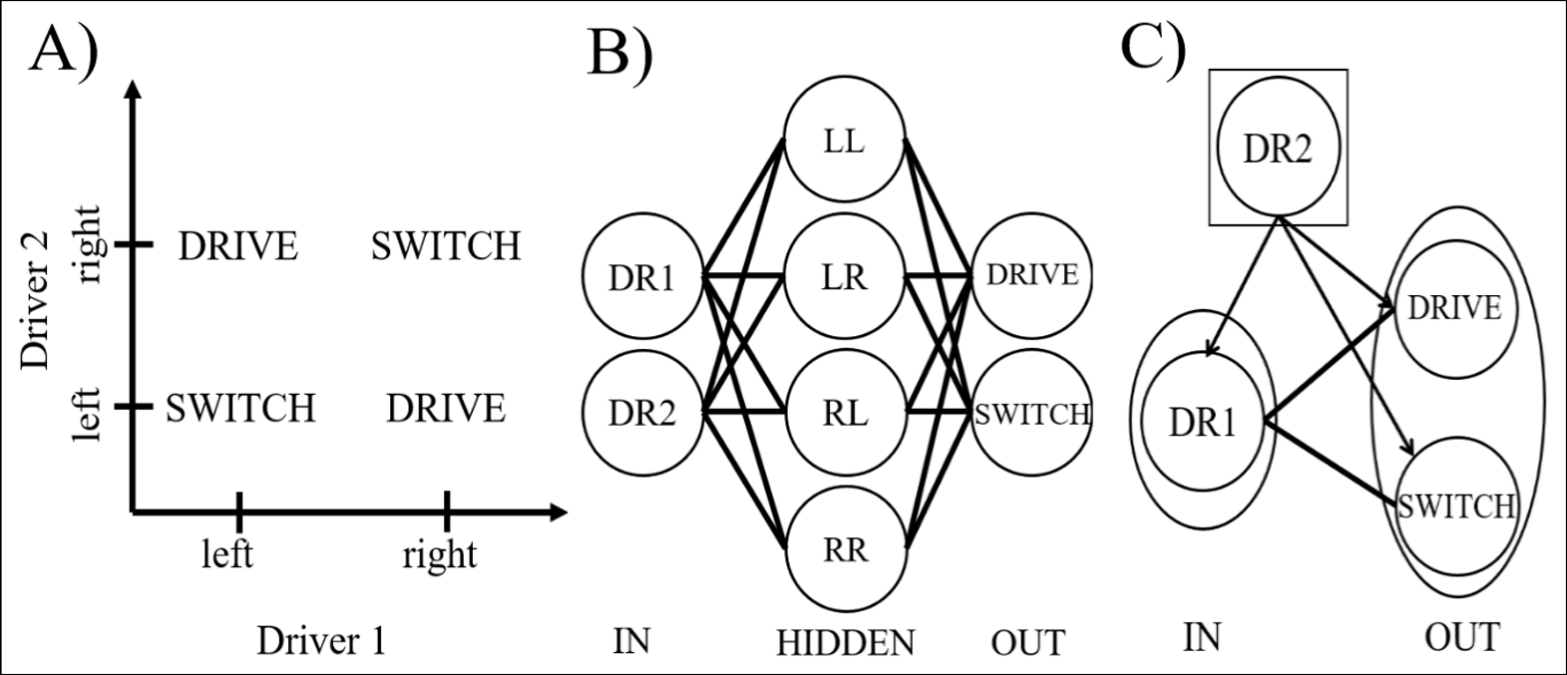
A depiction of a linearly inseparable problem, and possible computational solutions. **A)** A visualization of the input vector space of the bridge crossing problem with the possible states for driver 1 (DR1) on the x-axis and the possible states for driver 2 (DR2) on the y-axis. **B)** A model using flat binding. The two input units code for the lane each driver is using (left or right). The units in the hidden layers represent all possible options, going from both drivers driving in the left lane (top unit) to both driving in the right lane (bottom unit). The output units code for the possible responses: keep driving in your lane, or switch. **C)** A hierarchical binding model where the context determines the action taken by DR1. The context unit DR2 informs DR1 about the best possible course of action.

The ability to flexibly adjust behavior in response to a changing task environment is called cognitive control. It is widely acknowledged that cognitive and action control require binding elementary features together (e.g. Abrahamse et al., 2016; Frings et al., 2020). As a simple example, consider the problem of a car driver (driver 1) approaching a bridge. On the opposite side of the bridge, another car is approaching (driver 2). From the perspective of driver 1, the task is simple: If driver 1 drives on the right, and driver 2 drives on the left, then drive on; and if driver 2 drives on the right, then switch lanes. Opposite actions are in order if driver 1 drives on the left: See Figure 1A for a summary.

To solve this problem, perhaps the most straightforward approach that comes to mind is constructing appropriate synaptic connections (i.e., learn) between states (DR1 left, DR1 right, etc.) and actions (drive or switch) using a learning rule such as the Rescorla-Wagner (RW) learning rule (Rescorla & Wagner, 1972). However, it is well known that such a construction is impossible in this case. Because no straight line can separate the Drive from the Switch states in Figure 1A, this problem is technically called linearly inseparable, and a two-layered model is not sufficient to solve a linearly inseparable problem (Minsky & Papert, 1969). This problem can be solved, however, by introducing hidden units between states and actions (Figure 1B). Here, hidden units would represent all conjunctions between lower level features. For example, in the bridge crossing problem we have two drivers that each can drive either left or right yielding four different combinations. Each of these four combinations are represented by a separate dedicated unit in the hidden layer (Figure 1B). Because all units operate at the same level, we call this flat binding.

Besides flat binding, there is a different approach towards solving such problems, which we will call hierarchical binding (Figure 1C). In the bridge crossing problem, DR1 has two response options available, but the position of DR2 might serve as the context for choosing between driving on the same lane or switching. An example of hierarchical binding more familiar in experimental psychology, is the Stroop model of Botvinick et al. (2001). This model has (lower-level) word and color units, along with two (higher-level) task units coding for the relevant dimension (word/color) of the presented stimulus. These task units bias the lower-level units relevant to the task. For instance, when the task unit coding for color naming is active, it biases all color units. By biasing a subset of the lower level units depending on the task, hierarchical binding also allows solving linearly inseparable problems.

Besides learning synaptic connections, a second way to implement bindings is via coordination of oscillations (Hummel & Holyoak, 2003). At the neural level, strong evidence has been found for the involvement of oscillations at specific frequencies in binding (Engel et al., 1991; Fries et al., 2001; Womelsdorf et al., 2007). Especially theta (θ; 4 – 8 Hz) and gamma (γ; 40 – 80 Hz) have been suggested to be critical in learning and cognitive control (Cavanagh & Frank, 2014; Cohen, 2014b; Fries et al., 2007). Cavanagh and Frank (2014) postulate that θ is associated with the need for cognitive control. Canolty et al. (2006) demonstrated that high γ-amplitude (80 – 150 Hz) in the human medial frontal cortex (MFC) is modulated by θ-phase and that synchrony between θ and γ is task-dependent, suggesting that synchrony between specific brain rhythms is required to coordinate different brain areas to enable adaptive behavior.

Recently, Verguts (2017) unified these empirical findings in a computational model of cognitive control incorporating θ and γ neural oscillations. In this model, cognitive control is implemented via synchronization of task-relevant brain areas. Biologically speaking, when units oscillate in synchrony, their ion gates open and close simultaneously, allowing for optimal information sharing (Fries, 2015). In Verguts’ cognitive control model, synchronization between brain regions is achieved by simultaneously sending them θ-frequency (±6 Hz) locked bursts (Zhou et al., 2005). Thus, θ power is positively correlated with synchronization. As in Botvinick et al. (2001), this model implements hierarchical binding. Here, a higher-level module determines which lower-level units need to be synchronized (i.e. the higher-level module provides a context), while another higher-level module implements the synchronization. Through synchrony, task-relevant input exerts increased influence on the model output relative to task-irrelevant input, while earlier learned associations remain unaltered.

In the current paper, we explore how each approach to binding (learning and synchronization) impacts model performance. We accomplish this by testing four distinct models differing in their ability to learn and/or synchronize on three action control paradigms. Our models belong to the class of evidence accumulation models, and as such it provides two key behavioral metrics: reaction times (RT) and accuracy. But importantly, because it synchronizes neural modules, the model provides information on an additional (neural) metric: θ power. We will compare these metrics between our four models.

We manipulate the model’s ability to learn and synchronize on a trial-by-trial basis to assess how each component affects model performance. The ability to learn is controlled by altering the learning rate parameter in the model’s RW learning rule: in a non-learning scenario the parameter value is fixed at zero, while this value is set to an arbitrary value of 0.4 when learning can occur. When synchronization occurs, task-relevant input units are synchronized with the output units. If model synchrony is not allowed, this synchronization process does not take place. Thus, we have four different models depending on their ability to learn and synchronize. In summary, we investigate accuracy, RT and θ power fluctuations between four models differing on their ability to learn and synchronize.

The four models are tested on three distinct paradigms ranging in difficulty. In the first paradigm, the extensive learning paradigm, an agent learns simple stimulus-action associations through trial-and-error. Similar to the original paradigm (Huycke et al., 2021; Ruge & Wolfensteller, 2010), stimulus-action associations are repeated both within experimental blocks and between experimental blocks, allowing to investigate learning on a fast and a slow timescale respectively. The input vectors are orthogonal, which automatically renders the task linearly separable. Due to the orthogonality of the input vectors, this paradigm is considered to be the easiest of the three adopted designs. The second paradigm is a Stroop task (Stroop, 1935). In this paradigm, sixteen distinct stimuli (the combinations of four words and four colors) are shown to the agent. The goal is to learn which response (out of four options) is required for a certain stimulus. The input patterns are not orthogonal and the relevant dimension of the stimulus (word/color) is changed throughout the experiment several times, making this task linearly inseparable. The non-orthogonality of the input vectors also allows for catastrophic forgetting (McCloskey & Cohen, 1989) to take place. Catastrophic forgetting or catastrophic interference occurs when a model switches from one task (e.g. word naming in a Stroop task) to another task (in the example, color naming) and overwrites the weights learned in the initial task for the purpose of learning the second task, thus forgetting how to perform the initial task. Under catastrophic interference, the model is doomed to forget and relearn the task weights at every switch.

The final paradigm is the Wisconsin Card Sorting Task (WCST; Heaton, 1981). In this paradigm, an agent has to respond to one out of three stimulus dimensions: shape of the stimulus, number of stimuli shown, or the stimulus color. Each dimension has four possibilities. A total of 64 (4^3^) unique stimuli are seen by an agent performing this paradigm. Similar to the Stroop paradigm, the input patterns are not orthogonal and the relevant stimulus dimension is frequently switched during the experiment. Since the WCST is a generalization of the Stroop task, it is also linearly inseparable but more difficult than the latter. In summary, we test the performance of four models ranging in ability to learn and/or synchronize on three paradigms varying in difficulty and linear separability.

## Materials and Methods

**Figure 2:**
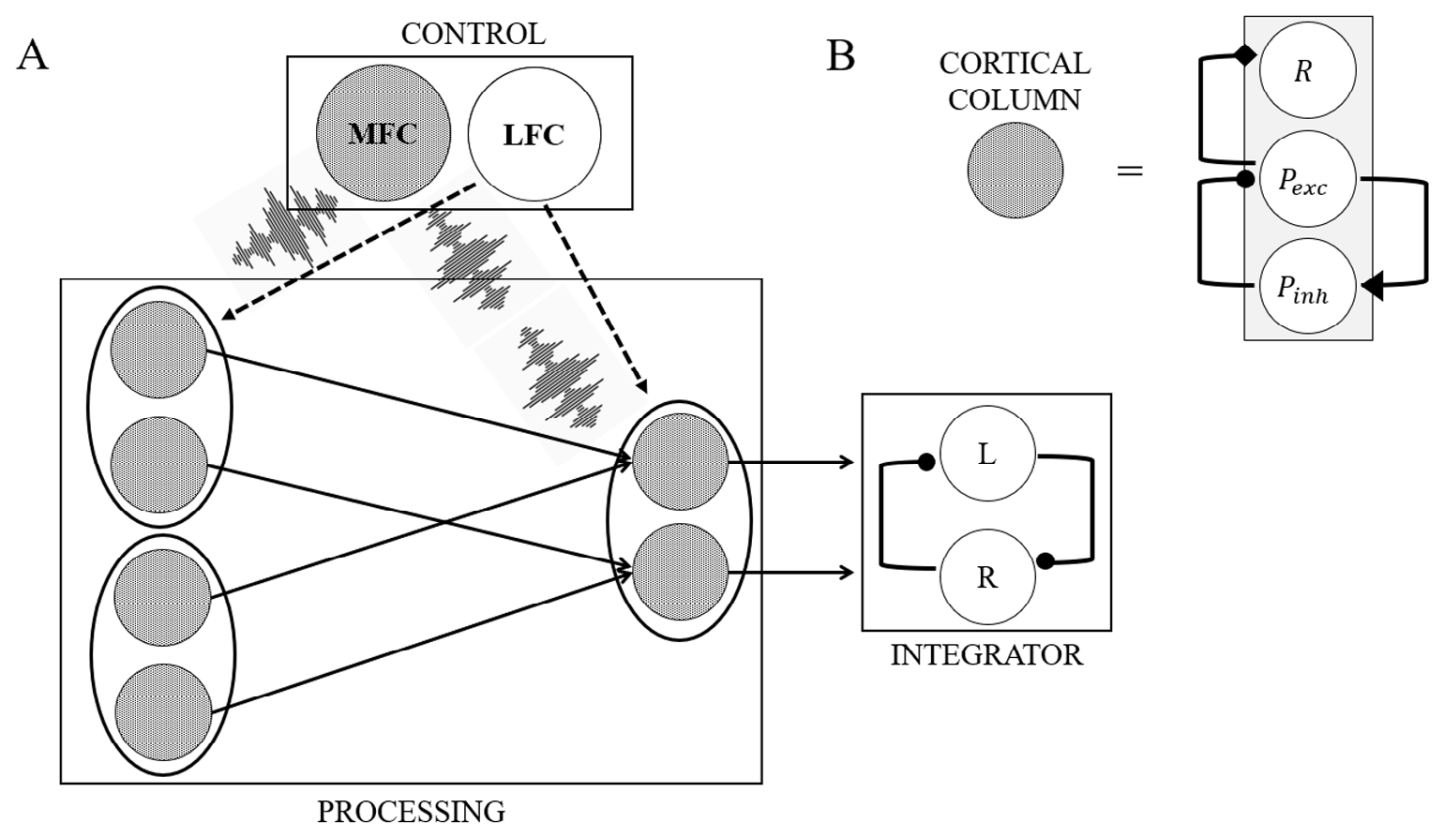
An example model displaying all modules, along with their units. **(A)** The control module (box top), a processing module (box left) with four input units (left) and two output units (right) and an integrator module (box right). The input units are fully connected with the output units via weights (arrows). During an experimental trial, the LFC might tag the first two input units for synchronization with the output units (dotted arrows), which is followed by synchronization of these tagged units via θ frequency-locked noise bursts sent by the MFC. **(B)** Illustration of a cortical column unit (grey). These units consist of one rate code neuron (R) and two phase code neurons (P). All cortical columns oscillate at a specific γ-frequency (46 Hz).

Although the models are manipulated in terms of their aim and abilities, they all share a common architecture. Namely, each model contains a processing module, an integrator module, and a control module (see Figure 2A), in line with earlier work describing similar computational models (Senoussi, Verbeke, Desender, et al., 2022; Verbeke & Verguts, 2019; Verguts, 2017). Each module is made up of model units. These units can be binary units or cortical columns. The binary units have only two states: on or off. Cortical columns (Figure 2B) are triplets consisting of one rate code neuron and two (one inhibitory and one excitatory) phase code neurons. The rate code neuron handles incoming information, while the phase code neurons coordinate the processing efforts of the rate code neuron by ensuring that information is sent to the correct processing area (Verguts, 2017). The phase code neurons are coupled via an arbitrary coupling parameter. We chose the value of this parameter so that all cortical columns oscillate at a particular γ-frequency (46 Hz).

The processing module consists of input units and output units, which are all cortical columns. Input units represent stimulus features, while output units represent potential responses. The processing module is fully connected, meaning that there is a weight between each pair of input and output units. During learning, weights between input and output units in the processing module are updated on a trial-by-trial basis via the RW learning rule (Rescorla & Wagner, 1972), with the aim to minimize the difference between the response given by the model and the desired response. Information is sent from the input units towards the output units in a feedforward fashion. The integrator module contains units which receive and accumulate information from the output units in the processing module. Whenever the accumulated activation of one of the units reaches an arbitrary threshold, a response is given by the model. The control module contains units representing the human lateral frontal cortex (LFC; containing binary units) and medial frontal cortex (MFC; containing cortical columns). This module implements synchronization: the MFC sends θ-frequency locked bursts to the units that are tagged by the LFC unit. Note that this model implements hierarchical binding: the control module allows biasing of specific input units allowing to solve linearly inseparable tasks without a hidden layer in the processing module.

### Learning

The weights in the processing module between input and output units are changed on a trial-by-trial basis according to the Rescorla-Wagner (RW) learning rule (Rescorla & Wagner, 1972). The RW learning rule adjusts weights with the goal to minimize the average error across trials. The following RW learning rule is used to update the weights in all our models:

\Delta {W_{io}} = \beta \times (Target - {X_o}) \times {X_i} \times {X_o}

Here, Δ*w_io_* represents the trial-to-trial weight change from an input unit *i* to an output unit *o, β* is the learning rate, *Target* represents the response that the model should give, *X_o_* denotes the actual response that is given by the model, and *X_i_* is the maximal activation across all timesteps in a trial for the input unit *i*. Note that the final term *X_o_* is not included in the original RW learning rule. We added this term based on earlier work (Verbeke & Verguts, 2019) to ensure that only units that are simultaneously activated are able to learn.

### Synchronization

We achieve flexible binding between model representations of stimuli and their associated actions via synchronization. Stimuli and actions are represented in a model as active input and output units, respectively. Since these units oscillate, efficient (Fries, 2015) communication between units arises when they are synchronized.

Binding is implemented by sending simultaneous bursts of noise to units, causing them to synchronize (‘noise induced synchronization’; Zhou et al., 2005). This process is regulated by two specialized units in the model: the MFC and the LFC. The MFC oscillates at θ-frequency (6 Hz). At each timepoint within a trial, the probability that the MFC will send a noise burst depends on the amplitude of this MFC θ oscillation, where a high amplitude signals a high probability that a noise burst will be sent. The amplitude of the MFC oscillation increases within a trial, meaning that more synchronization events take place as trial time progresses until a unit in the integrator module reaches a threshold and a response is given. When the threshold is reached, the MFC oscillation is slowly drawn towards a near-zero value, decreasing the likelihood that synchronization events take place. Thus, the MFC determines when synchronization takes place. The LFC holds one binary unit for each stimulus or action collection used in our tasks (e.g., all word labels in the Stroop task would be considered as one stimulus collection). When a particular stimulus or action collection is task-relevant, its corresponding LFC unit is set to 1. To implement synchronization, the MFC signal is multiplied (thus implementing multiplicative gating; Verbeke & Verguts, 2022) with this LFC unit allowing synchronization. Hence, LFC determines which processing module units are synchronized. We measure θ power in a trial by squaring the amplitude of the MFC θ-oscillation within a trial. We refer the reader interested in an in-depth explanation of the synchronization principle to the original paper describing this theory (Verguts, 2017). In summary, synchronization is implemented through the interplay between the MFC and LFC, and enables a model to flexibly bind information in a neuronally plausible way.

## Simulations

To assess how the ability to learn and/or synchronize influences model performance, we trained and tested four models (No Sync/No Learn, No Sync/Learn, Sync/No Learn, Sync/Learn) on three action control paradigms. The two models that are unable to learn serve as a baseline for the other models, since the non-learning models are expected to perform at chance level. To assess the impact of learning and synchronization on model performance, we compare reaction times (RT), accuracy and θ power between different models, averaging across simulations.

Specific model parameters were changed depending on whether learning or synchronization could be used. In learning and non-learning models, the learning rate β was set to 0.4 and 0, respectively. For synchronization, we included a parameter that indicated whether the activation in the MFC unit (in terms of amplitude) was allowed to build up or not within a trial over time. Since the probability that noise bursts are sent depends on the amplitude of the θ-oscillation in the MFC, we manipulate the occurrence of synchronization via this parameter. Apart from the learning/synchronization manipulation, all other model parameters remained constant both within and across paradigms.

In the Stroop model, the weights were hard coded prior to the learning phase to ensure that the model performed better at word naming, in line with the empirical prepotency of reading words over naming colors (Stroop, 1935). All other models started with a random weight configuration near zero. Each trial lasted for a maximum of 500 timesteps, where a response was issued when one of the units in the integrator module reached an arbitrary threshold. The timepoint this threshold was reached is the RT (e.g. Ratcliff, 1978). When the threshold was reached, the most active output unit reflected the model’s response. We computed accuracy by comparing the model’s response with the target response (i.e. the predetermined correct response for this specific trial). When the threshold was not reached, this signaled that the model was unable to make a choice. The model’s θ power prediction is computed by squaring the amplitude of the MFC θ-oscillation.

The model scripts and accompanying simulation code were custom-made and written in Python 3. Subsequent visualization was done in R (R Core Team, 2020) using ‘ggplot2’ (Wickham, 2016), and in Python 3 using ‘seaborn’ (Waskom, 2021).

## Study 1: Extensive Learning

### Paradigm

The first paradigm is based on an extensive learning paradigm (Huycke et al., 2021; Ruge & Wolfensteller, 2010) where human subjects learned arbitrary stimulus-action associations through trial-and-error. Four stimuli are shown in each experiment block. Two of these are associated with a right response, the other two are associated with a left response. The agent must learn the correct stimulus-action mapping for each stimulus in an experimental block. Within a block, each stimulus is shown eight times, resulting in a total of 32 trials per block. This repetition of stimuli within an experimental block is labeled the ‘fast timescale’. In each experimental run, one stimulus collection is repeated across experimental blocks, with their stimulus-action mapping remaining constant. This repetition of stimuli is called the ‘slow timescale’. Of the 16 blocks performed in total, half contained a repeating mapping, and the other half were novel mappings. This repetition of stimulus-action associations across experimental blocks is named the ‘slow timescale’. In this paradigm, an agent sees 36 (4 + 4 × 8) unique stimuli, spread across 512 trials (32 × 16). Our models train on the trials that we presented to our 29 human participants (Huycke et al., 2021). Thus, for each subject who completed an experimental run, we let our four models train on exactly the same task. This means that the presentation order of the stimuli, which stimuli were shown, which stimulus set was repeated etc., were all shared between a certain subject and their model counterparts. Since we have four different types of models based on the presence of learning and synchronization we have a total of 116 (4 × 29) different models.

### Model overview

**Figure 3:**
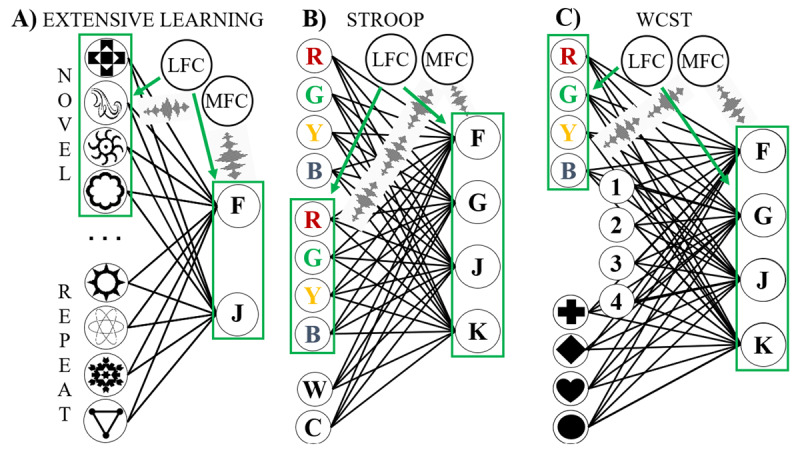
An overview of the three paradigm-specific models. The integrator unit is omitted for visualization purposes. **A)** Extensive learning model. Each of the 36 input units (left; only 8 units depicted for clarity) represents a stimulus. The output units represent the possible actions. In this example, the first four input units are synchronized with all output units via an interplay between the LFC and MFC. **B)** Stroop model. Input units 1 – 4 represent the written word, units 5 – 8 represent the word color, and units 9 – 10 represent the relevant stimulus dimension. The output units represent actions. In the panel, all color units are synchronized with all output units. **C)** WCST model. Input units 1 – 4 code for stimulus color, units 5 – 8 represent the number of shapes shown, and units 9 – 12 code for stimulus shape. The output units again represent possible actions. Here, the MFC and LFC cooperate to synchronize all color units with the output units.

The processing module of the extensive learning model contains 36 input units and 2 output units (Figure 3A). Each input unit represents a single stimulus, while the output units represent the left and right responses. On each trial, only the input unit coding for the presented stimulus is active. When the model is initialized, the weights between the input units and the output units are randomly drawn from a uniform distribution between 0 and 0.1. When learning takes place, these weights are altered on a trial-by-trial basis. All other weights are initialized with a value of zero and remain constant throughout the experiment. When synchronization is implemented, the LFC unit tags (i.e., multiplies, see description above) input units representing stimuli belonging to the same experimental block for synchronization with the output units. The architecture of this model, along with a visualization of the synchronization process, appears in Figure 3A.

### Results and Discussion

We investigate how RT, error rate and θ power evolve in the extensive training model by plotting their mean values and associated 95% confidence intervals (CI). Because we were mainly interested to see which of the four models could qualitatively fit the behavioral (RT & error rate; Anderson, 2013; Fitts, 1967; Snoddy, 1926), and neural (θ power; Clarke et al., 2018; Crivelli-Decker et al., 2018) data patterns reported in the literature, we did not perform a systematic model fitting procedure. Thus, when we say that one model “outperforms” some other, we mean that the former qualitatively captures the empirical data patterns (more details follow in the specific model discussions).

Additionally, we tested the impact of the specific MFC θ frequency. In the results reported below, we set coupling parameter *C* to 0.07 (leading to an MFC oscillation frequency of 5.57 Hz), but our results are qualitatively very similar when the MFC had different frequencies (*C* = 0.05, oscillation at 3.97 Hz; or instead *C* = 0.10, oscillation at 8 Hz). Hence, the specific θ frequency at which the MFC unit oscillates did not impact our results.

Starting with the results on the fast timescale, we plotted the simulated data as a function of how often a stimulus was presented in an experimental block, from here on referred to as stimulus number. We looked at data where the total number of times a stimulus was seen was less than 9, meaning that only novel data and the first block of the repeating data were visualized for the fast timescale. The comparison between the four models is most relevant in the later, linearly inseparable tasks (Stroop and WCST), as this is where we expect to see the added benefit of synchronization. Since the current paradigm is linearly separable, we expect no difference and hence we only visualize results for the most complex (Sync/Learn) model in the main text. We refer the reader to Figures S1, S2 and S3 for results on the other three models.

**Figure 4:**
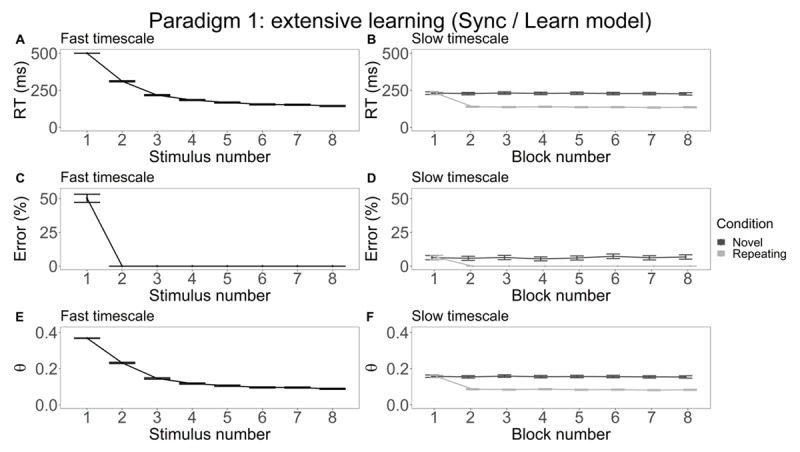
Extensive learning simulation results for the Sync/Learn extensive training model. The left column (panels A, C and E) contains results for the fast timescale, whereas the right column (panels B, D, and F) focuses on the slow timescale. The first row (panels A and B) display RT results, the next row shows the number of errors that the models made on average (panels C and D), and the final row (panels E and F) visualizes how θ power fluctuates on each timescale.

RT decreases significantly as a function of stimulus number (Figure 4A). More precisely, RT decreases significantly with each extra stimulus encounter until the sixth repetition. The decrease in RT is strongest for the first three stimulus repetitions. For error (Figure 4C), we see a similar effect of experience: The first time a stimulus is shown, the model responds at chance level (50%), but for every subsequent stimulus repetition the model makes no errors. Finally, we turn to θ power (Figure 4E). We note that θ power decreases as a function of stimulus number. Similar to RT, the observed decrease in θ power is strongest for the first three repetitions and reaches an asymptote at stimulus repetition six. On the fast timescale, the Sync/Learn model significantly outperforms the non-learning models (No Sync/No Learn and Sync/No Learn) on all metrics. Interestingly, no significant differences were observed between the Sync/Learn model and the No Sync/Learn model (Figure S3; panels A, C and E).

**Figure 5:**
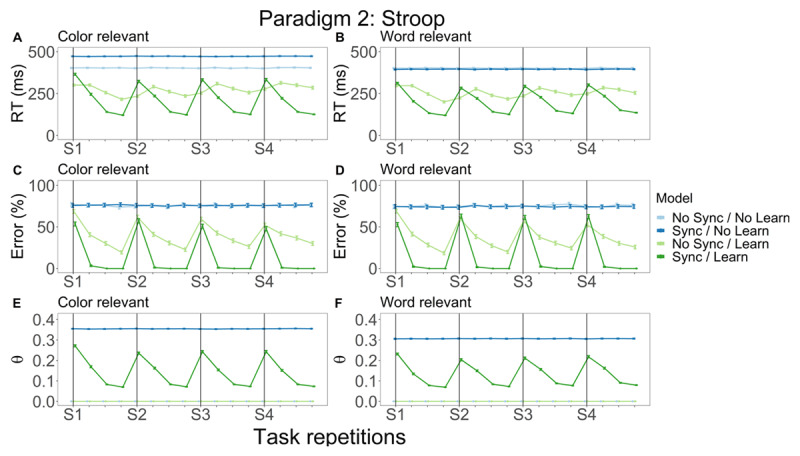
Stroop model simulation results: RT, error rate and θ power. Different colors represent distinct models while columns denote the relevant stimulus dimension. In the left column, we plot RT **(A)**, error **(C)** and θ power **(E)** as a function of task repetitions for trials where the color dimension was relevant. In the right column, RT **(B)**, error **(D)** and θ power **(F)** were visualized, but now for trials where the word dimension was relevant. Green lines reflect learning, dark colors indicate that synchronization took place.

For the slow timescale, we plotted the same metrics as a function of block number. On this timescale, we make a distinction between the novel and the repeating condition where novel blocks contain stimuli unique to that specific experimental block and repeating blocks contain the repeating stimulus set. Average RT, error rate, and θ power as a function of block number are depicted in Figures 4B, 4D, and 4F respectively. Inspecting simulated RT on the slow timescale (Figure 4B), we remark that average RT remains constant across experimental blocks for the novel condition. In the repeating condition however, average RT is significantly higher in block 1 compared to the following blocks. This dynamic is also seen for error (Figure 5D) and θ power (Figure 5F). In line with the results on the fast timescale, the Sync/Learn model is significantly faster and more accurate than the non-learning models. Similar to the fast timescale, we see no significant differences between the Sync/Learn model and the No Sync/Learn model (Figure S3; panels B, D and F).

We will first compare the simulation results between models before turning to the comparison between timescales. We note an effect of learning but no effect of synchronization. Comparing behavioral results between the non-learning models (No Sync/No Learn and Sync/No Learn) and the learning models (No Sync/Learn and Sync/Learn), we see faster and more accurate responding with practice across timescales only for learning models. Apart from the influence on the behavioral metrics, the ability to learn also modulates simulated θ power. In the θ power metrics of the Sync/Learn model, we see that θ power decreases with stimulus number. Since θ power is related to RT (where faster RT means that fewer synchronization events take place), this decrease in θ power related to the decrease in RT is expected. Thus, these results suggest that learning significantly impacts both the behavioral and neural metrics. To investigate the effect of synchronization on model performance, we compare the behavioral results of the No Sync/Learn model with the results of the Sync/Learn model. No significant difference in behavioral performance between is found between these two models, showing that the ability to synchronize does not impact model performance. This is expected given the orthogonality of the input vectors in this paradigm: a model benefits from synchronization when it helps in overcoming the negative effects of catastrophic forgetting (McCloskey & Cohen, 1989) by protecting already changed weights and boosting communication between relevant input units and the model’s output units. Since the extensive learning task has orthogonal inputs, catastrophic forgetting does not occur, so the ability to synchronize provides no additional benefits. In summary, learning modulates both the behavioral and neural metrics, but synchronization has no significant impact on behavioral model performance in the current experimental paradigm.

Since the No Sync/Learn model and the Sync/Learn model are not significantly different on the behavioral level and only the latter model has θ power predictions, we interpret the comparison across timescales from the viewpoint of the Sync/Learn model. In the fast timescale, we see a decrease across all recorded metrics as a function of stimulus repetitions. In particular, the Sync/Learn model becomes faster, more accurate and shows less θ power as it gains more experience with a stimulus. The behavioral metrics again point to a practice effect, while these θ power dynamics might again indicate that less synchronization takes place as RT decreases due to continued learning. On the slow timescale, we see that the recorded metrics remain constant as a function of block number for the novel condition. This is expected, since this condition considers blocks where a new stimulus-action mapping has to be learned. Since this process is the same in each separate block, we expect no performance differences between the experimental blocks. In the repeating condition, we see a significant drop across all metrics when comparing the first experimental block with the subsequent blocks. Here, the first block represents the model learning a new stimulus-action mapping, whereas the subsequent blocks implement a repetition of the mapping learned in the first block. Hence, it is expected that the model is faster, more accurate and exhibits lower θ power from block 2 onward. We note that there is no significant difference between the first blocks of the different conditions, indicating in both conditions the first block is used to learn a new mapping. The significant difference between the following blocks indeed indicates that a well-known mapping recurs in the repeating condition. In summary, the comparison between the fast and the slow timescale for the Sync/Learn model indicates that learning takes place both on the fast timescale and for the repeating condition of the slow timescale.

## Study 2: Stroop

### Paradigm

In our version of the Stroop task, one of four words is presented in each trial (RED, GREEN, YELLOW and BLUE). This word is printed in one of these four different colors. Combining these features yields 16 distinct stimuli, where 25% is congruent (e.g., RED printed in red) and the other 75% is incongruent (e.g., RED printed in blue). The model has to respond to either the word dimension or the color dimension, choosing between four actions. We define an experimental block as a collection of the 16 unique stimuli. In half of the simulations, the model starts with word naming while in the other half the model starts with color naming. Each task is performed for four consecutive blocks before the task is changed and again performed for four blocks in a row. This dynamic is alternated four times, resulting in a total of 32 training blocks and a total of 512 trials per model. We let 100 agents model train on the Stroop task, leading to 400 distinct datasets.

### Model overview

The Stroop model’s processing module consists of ten input units and four output units. Input units 1 – 4 represent the written word shown to the model. Input units 5 – 8 represent the color the stimulus is printed in. The final two units of the input layer (task units) code for the relevant stimulus dimension (word or color). The four units of the output layer represent the four possible responses (Figure 3B). On each trial exactly three input units are active. The input and output units are connected using weights. We bias the weights from the word units to the output units to reflect the empirically observed advantage of word naming over color naming seen in human subjects (Stroop, 1935). This is implemented by assigning a larger positive numerical value to these weights when initializing the model. As a result, the Stroop effect naturally emerges.

This model implements flexible stimulus-action binding via the control module. Depending on the task unit activation, specific input units are selected for synchronization with the output units. For example, when the color task unit is active, the color units in the processing module are tagged by the LFC for synchronization by the MFC. These units will then be synchronized with all output units. This ensures that the color units, even though their weights are relatively weaker, can communicate more effectively with the output units theoretically resulting in improved performance. This model, and the binding of the color units with the output units, are depicted in Figure 3B.

The Stroop task does not have orthogonal input vectors, which implies that catastrophic interference will take place when the relevant task dimension is switched. Specifically, each subtask is linearly separable on its own, but switching between these tasks makes the Stroop task linearly inseparable. Synchronization of the relevant input units with the output units mitigates the catastrophic forgetting (McCloskey & Cohen, 1989) taking place after a task switch by biasing information processing in favor of the relevant stimulus dimension. Thus, rather than relearning the weights every time the relevant dimension changes, synchronization allows the weights for each dimension to remain intact.

### Results and Discussion

We visualized RT, error rate and θ power as a function of task repetitions, which can be conceptualized as experience with a certain stimulus dimension (word/color). In the plots, we distinguish between the two different relevant dimensions. Different models are represented by different colors.

We plot RT (Figure 5A) and error rate (Figure 5C) as a function of task repetitions for trials where color was the relevant stimulus dimension. Both non-learning models show a constant RT and error rate throughout the experiment indicating that for these models behavioral performance does not improve as a function of practice. An effect of learning is seen when comparing behavioral metrics between the No Sync/Learn (light green line) and the Sync/Learn models (dark green line): RT and error rate both decrease as a function of practice until a switch (S) in relevant dimension occurs. Although the general dynamics are similar, we see marked differences between the learning models, whereby the Sync/Learn model outperforms the No Sync/Learn model. When the relevant task dimension switches in the No Sync/Learn model, RT remains initially low (but accompanied by low accuracy) before increasing again in the second block, after which it again gradually decreases. This model shows decreases in error with more experience, but even after four blocks where the same stimulus dimension is relevant this model still performs relatively poorly (approximately 20% errors). In the Sync/Learn model, RT and error rate are significantly higher and lower respectively after switching relative to the No Sync/Learn model. Both metrics also decrease significantly faster as practice takes place. The Sync/Learn model is significantly more accurate than its No Sync/Learn counterpart. Finally, we consider θ power. Since no θ power can be observed if synchronization is not implemented, we limit our discussion to the Sync models. In Figure 5E, we see that θ power remains constant for the Sync/No Learn model throughout the experiment. For the Sync/Learn model, we see that θ power decreases as a function of task repetition similar to the decrease seen in RT (Figure 5A) showing the relationship between faster RT and lower θ power.

Next, we consider the trials on which the word stimulus dimension was relevant. RT (Figure 5B) is significantly lower for the non-learning models when comparing to RT for these models in the color condition. Similarly, for the Sync/Learn model, RT is also significantly lower when compared to the color condition. No differences are observed for error rate (Figure 5D). Finally, θ power (Figure 5F) is significantly lower for both Sync models relative to the color condition. Otherwise, results were very similar to those in the color dimension.

We observed effects of both learning and synchronization. Similar to the extensive learning paradigm, the effect of learning appears when comparing the non-learning with the learning models. Only the learning models improve behaviorally as a function of practice. Considering RT in the learning models, RT is generally high when a new task is encountered but subsequently decreases as more task experience is gained. This dynamic is very apparent for the Sync/Learn model, but more attenuated for the No Sync/Learn model. A potential explanation as to why RT in the No Sync/Learn remains relatively constant throughout the experiment is because the magnitude of the weights (sum of the squared weights; technically, the norm of the weight matrix) does not change drastically throughout the experiment. Since the norm of the weight matrix is similar throughout the experiment, the model will have a relatively constant RT, but error rate (which depends on the structure of the weight matrix) still fluctuates. Error rate is high at first but decreases as the models practice responding to a certain stimulus dimension. Although error rates are similar for the two learning models, we see significant differences between the No Sync/Learn model and the Sync/Learn model across conditions. The difference could be attributed to the advantage of word naming implemented when creating the Stroop model. Even though there are some condition-specific differences, overall error rate in the No Sync/Learn model decreases significantly more slowly than for the Sync/Learn model. Additionally, error rate decreases to 0% with more practice for the most complex model while this is not the case for the No Sync/Learn model. Taken together, these results indicate that synchronization boosts accuracy in this paradigm. This is expected: synchronization mitigates catastrophic forgetting after switches in the relevant stimulus dimension by protecting the weights and biasing information processing to elevate the impact of relevant input units on the model’s output. Thus, the significantly lower error rates in the Sync/Learn model can be attributed to the effects of synchronization. Finally, we consider θ power in the Sync/Learn model. Similar to the results for the extensive training paradigm, θ power shows similar dynamics to RT. Specifically, when the relevant stimulus dimension is changed, both θ power and RT initially increase but subsequently decrease as a function of experience. These results again indicate that θ power decreases due to the faster RT, which in turn is an effect of learning. In summary, the model comparison indicates that the combination between learning and synchronization allows optimal performance based on the resulting RT and accuracy measurements.

As a final point, the model is faster and more accurate when the word dimension is relevant. This trend is expected given the model’s architecture: A bias in weight strengths in favor of word naming was implemented to reflect (literate) humans’ prepotency for reading words (Stroop, 1935).

## Study 3: Wisconsin Card Sorting Task

### Paradigm

The final paradigm we consider is the WCST. At an abstract level, the WCST can be seen as a generalization of the Stroop task, where the agent has to respond to one out of *N* > 2 stimulus dimensions. In our version of the WCST, there are three different stimulus dimensions: the color of the stimulus (red, green, yellow, blue), the number of stimuli that are shown (1, 2, 3, 4), and the shape of the stimulus (cross, diamond, heart, shape). Since for each of the three stimulus features four different options are available, 64 (=4^3^) unique stimuli are seen by the model. In line with our approach of the Stroop model, we define an experimental block as a collection of these 64 unique stimuli. The training of the model starts with three blocks where color is the relevant dimension. In the next three blocks the number of stimuli is relevant. In the following three blocks, the stimulus shape is relevant. This order of blocks is repeated four times, leading to 36 blocks in total, and 2304 (64 × 36) training trials. Similar to the Stroop model, we run 100 simulations for each of the model types, resulting in 400 unique datasets.

### Model overview

The WCST model is shown in Figure 3C. The processing module consists of 12 input units and 4 output units. Input units 1 – 4 represent the color dimension of the stimulus. Input units 5 – 8 represent the number of stimuli that are shown. The final four input units represent the shape of the input. We highlight that, similar to the Stroop model, three input units are active when a stimulus is presented. The four output units represent the possible actions. The input layer is connected with the output layer via weights. A control module consists of an MFC and LFC. Depending on the relevant stimulus dimension, the units coding for this stimulus dimension will be synchronized with all the output units.

The architecture of the input layer is similar to that of the Stroop model, with three major differences. The first difference is the difficulty: in the Stroop task, only one dimension (e.g. color) interferes with the relevant stimulus dimension (in the example, word), whereas in the WCST two irrelevant dimensions (e.g. number, shape) cause interference (with color). Another difference is that the WCST model lacks task units. Which input units need to be tagged for synchronization by the LFC is hard-coded in the WCST model without the need for extra units, whereas the Stroop model could determine the relevant dimension based on the activation of two dedicated task units. The final difference is the level of incongruency experienced during a trial. In the Stroop task, stimuli are either congruent (word = color) or incongruent. In the WCST, trials can have three levels of congruency. For example, consider a trial where the model has to respond to the color dimension. If a stimulus is presented where all dimensions point to the same (correct) answer, the model will experience no incongruency. When two stimulus dimensions (e.g. color and number) point to the correct answer but the third stimulus dimension (e.g. shape) points to the incorrect answer, a medium amount of incongruency arises. When both other stimulus dimensions (number and shape) point to an (incorrect) answer, then a high amount of incongruency arises.

Since the WCST is a generalized version of the Stroop task, this task is also not linearly separable. We again expect that synchronization can compensate model performance when a switch in relevant stimulus dimension occurs. Additionally, model performance is impacted by the level of incongruency that is experienced during a trial. When no incongruency is experienced, a model is hypothesized to respond faster and more accurately than when two out of three features point to the same but incorrect response. Synchronization may also improve model performance by biasing information processing towards the underrepresented but relevant stimulus dimension, overcoming the incongruency experienced by the model.

### Results and Discussion

We plot RT, error and θ power as a function of task repetitions. In this paradigm, each stimulus dimension is relevant for three experimental blocks in a row. Then, another stimulus dimension becomes relevant again for three consecutive blocks. We visualize our results accounting for incongruency, showing results for the lowest and the highest levels of incongruency. No incongruency means that all three stimulus dimensions point to the correct response. The highest level of incongruency means that the two irrelevant stimulus dimensions point to a different incorrect response. For the sake of brevity, we do not discuss the medium congruency plot and the high incongruency plot pointing to the same response in the main text, but they appear in Figure S4. As before, we also plot individual results for each of the four models.

**Figure 6:**
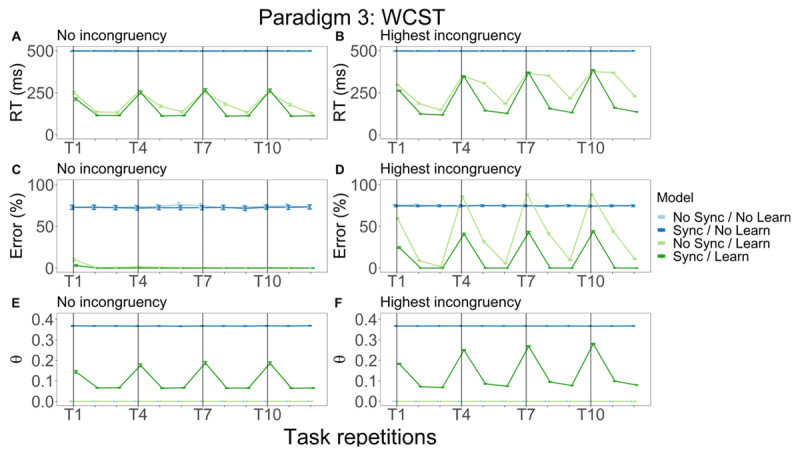
WCST model simulation results: RT, error rate and θ power. Different colors represent distinct models while columns denote the incongruency level. In the left column, we plot RT **(A)**, error **(C)** and θ power **(E)** as a function of task repetitions, for trials without incongruency. In the right column, RT **(B)**, error **(D)** and θ power **(F)** are visualized for the highest incongruency trials.

For trials without incongruency, RT (Figure 6A) remains constant at the maximum for the non-learning models throughout the experiment. For the No Sync/Learn model (light green line), RT starts at a high value, but decreases with more practice. When the relevant dimension is changed, an initial increase is followed by a decreasing average RT with practice. Similar dynamics are seen for the Sync/Learn model (dark green line), but here, RT values are significantly lower relative to the No Sync/Learn model. The non-learning models constantly perform at chance level (75% error rate). The No Sync/Learn model shows a small error rate in the first experimental block, followed by a zero percent error rate in the rest of the experiment. The Sync/Learn model starts with a significantly lower error rate, and also makes zero mistakes in the subsequent experimental blocks. By design, we observe no θ power for the No Sync/No Learn and No Sync/Learn models (Figure 6E). Similar to the Stroop results, θ power remains constant for the Sync/No Learn model. For the Sync/Learn model, θ power starts high, decreases in the second block and remains constant in the third block. This dynamic is observed for every following switch in relevant dimension.

RT (Figure 6B) and error rate (Figure 6D) at the highest level of incongruency again remain constant at the maximal value for the non-learning models throughout the experiment. For the No Sync/Learn model, both RT and error rate start at a high value, but decrease with more practice for the first stimulus dimension. After the switch, we see that both metrics remain relatively high in the next block before they drop in the third block. This dynamic is repeated for later relevant stimulus dimensions. Interestingly, when a switch occurs, error rate is worse than chance level before it drops with training. This dynamic repeats for later repetitions. The error rate significantly increases across time. In the Sync/Learn model, RT is high when the switch is made, but quickly drops to an asymptote where it remains until the relevant stimulus dimension is switched. The Sync/Learn model again starts with a significantly lower error rate and achieves a zero error rate with more training. Similar to the No Sync/Learn model, error rate increases with a switch in relevant dimension, albeit significantly lower. Turning to θ power, we do not observe this metric for the No Sync/No Learn and No Sync/Learn models (Figure 6F). Similar to the Stroop results, θ power remains constant for the Sync/No Learn model. For the Sync/Learn model, θ power starts high, decreases in the second block and remains constant in the third block. This dynamic is observed for every following switch in relevant dimension.

Overall, results are similar to the Stroop model: constant suboptimal performance for non-learning models, and decreasing RT, accuracy and θ power values as practice takes place for learning models. In line with the Stroop results, we again observe a beneficial effect of synchronization on the behavioral metrics due to the linear inseparability of the WCST. This difference is mainly visible when comparing the significantly faster and more accurate results for Sync/Learn model when comparing with the No Sync/Learn model. This comparison suggests that behavioral performance is aided by synchronization as this dampens the effects of catastrophic forgetting that take place whenever the relevant stimulus dimension is switched. In short, the between-models comparison corroborates the finding from the Stroop paradigm that the combination of learning and synchronization allows for optimal model performance when the task is linearly inseparable.

## Discussion

In this study, we compare simulated behavioral and neural metrics of four computational models differing in ability to learn and to synchronize across three action control paradigms: an extensive learning task, a Stroop task, and the WCST. The ability to learn consistently has a positive impact on behavioral model performance, as evidenced by faster RT and more accurate responses as a function of practice in learning models as compared to non-learning models. Additionally, we report a selective beneficial effect of synchronization on performance: a learning and synchronizing model performs faster and more accurate as a function of practice than the learning-only model for linearly inseparable tasks (Stroop, WCST) but not for the linearly separable one (extensive learning), in which case the two learning models performed similarly. At the neural level, we report decreased θ power as a function of practice in all paradigms. We also show that simulated θ power is affected by the ability to learn: θ power follows the changes in RT that take place over the course of learning. This is intuitive considering how synchronization is implemented in our models: The probability that a synchronization event takes place increases with trial time. Thus, the slower the model RT, the more synchronization events have taken place in that trial. Taken together, these results suggest that learning is essential for behavioral improvement; and model performance benefits from synchronization depending on the task requirements.

In this work, binding is achieved through learning and synchronization but different theories concerning binding are proposed in the literature. It is particularly worthwhile pointing out the relationship with the Binding and Retrieval in Action Control (BRAC; Frings et al. (2020)) framework. Generalizing the notions of an object file (Kahneman et al., 1992) and ideomotor learning (James et al., 1890), Hommel et al. (2001) proposed in their Theory of Event Coding (TEC) that stimulus, response and outcome features are bound together into a single episodic entity labelled an ‘event file’. BRAC can be considered as another generalization of this theory, focusing on the applicability of the framework across several experimental paradigms. BRAC identifies two major processes implicated in action planning: binding and retrieval. In our models, the fast binding in the sense of BRAC, at the time scale of a single experimental trial, is accomplished via θ oscillations. However, another, slower, type of binding exists, namely learning (synapses) between processing units. The latter type of learning was instantiated in our models via RW learning (Rescorla & Wagner, 1972). Both types of bound information are retrieved in the same way in our models.

Our model suggests to explicitly consider hierarchical binding in BRAC; for example, outcomes may provide a context to bind different stimuli and actions, depending on current task demands. An advantage of such an hierarchical approach is that knowledge is transferred more easily to new environments (Badre et al., 2010; Higgins et al., 2016) since information is stored in a modular fashion. Consistently, humans can easily transfer knowledge to new contexts (Badre et al., 2010; Franklin & Frank, 2020; Vaidya et al., 2021). Our results additionally show that hierarchical binding allows an agent to perform optimally in a complex environment.

Following the principles introduced in TEC (as mentioned above, the predecessor of BRAC), Haazebroek et al., (2017) already developed a hierarchically organized computational model on the interaction between perception and action control: HiTEC. In HiTEC, task units modulate representations at the lower (perception and action) model levels. Similarly, in our models a task unit (i.e. the LFC unit) provides information about the task at hand and tags relevant model units for synchronization. Besides hierarchy, a second similarity between HiTEC and our models, is that both models learn associations via contingency learning. In HiTEC, associations are learned via Hebbian learning (Hebb, 1949), whereas in our model stimulus-response associations are learned through RW learning (Rescorla & Wagner, 1972).

Now focusing on differences between HiTEC and our models, we note that HiTEC incorporates so-called feature units, which code for abstract features (e.g., “redness” irrespective of the input modality). Such feature units allow for the implementation of the ideomotor principle on which the theory was originally based. For example, when a motor code produces a certain perceivable (haptic) effect, a connection is formed between the haptic sensation units that were activated as a result of the motor act, and a feature node that originally led to the motor activation. As a result, later thinking about the (haptic) effect (ideo-) may activate the feature unit, which itself can activate the corresponding motor unit. In our models, there are no feature units, but this is merely a practical, not a theoretical choice: It will be interesting to explore the inclusion of feature units in our models in future work. As a second difference, our sync model but not HiTEC uses neural oscillations for fast bindings. As mentioned, oscillations are computationally efficient and empirically valid, as confirmed in several earlier studies (Cohen, 2014a; Senoussi, Verbeke, & Verguts, 2022; Voloh & Womelsdorf, 2016; Womelsdorf & Everling, 2015).

In an effort to relate neurophysiological measures to the formation of event files, Takacs and colleagues (2020) analyzed EEG data recorded during an event file coding paradigm (Hommel, 1998). Using EEG and network analysis, the authors showed that event file binding processes were reflected by changes in θ band network architecture. More precisely, a different level of small-worldness (see Takacs et al. for details), was observed in the brain network in trials where less versus more binding was required (again, see Takacs et al. for details). Following these results, one might argue that θ band modulations should be seen mainly in terms of (small-world) network characteristics, and not in power fluctuations as proposed here. However, these results are difficult to compare with our results since their paradigm and analysis approach are so different from ours. Specifically, the Takacs et al. paradigm was designed to investigate interactions between stimulus feature overlap and responses, and it was for this specific interaction that no effect for θ power was observed. In contrast, our paradigm focused on extensive learning: agents had to learn for a prolonged period which stimulus is associated with which response. Instead, no learning takes place in the event coding paradigm. To more tightly match the current model and the earlier data, it will be imperative in future work to calculate network properties (such as small-worldness) on our sync models; and investigate θ (power) fluctuations when the event file coding task is administered in an extensive learning paradigm.

Switching the relevant stimulus dimension increases RT and error rate in the Stroop and WCST models, suggesting that these models experience a switch cost. The switch cost is well-documented (Kiesel et al., 2010; Koch et al., 2010), and is captured by two prominent theories (Vandierendonck et al., 2010). The first one (interference theory; Allport et al., 1994) views a switch cost as a continuing interference from earlier activated task-set features. The second one (reconfiguration theory; e.g. Monsell & Mizon, 2006). views the switch cost as resulting from the reconfiguration of the novel task set. As already noted by Vandierendonck et al. (2010), the two frameworks can be integrated, and in line with this view, the current modeling framework combines aspects of both theories. When a switch occurs, the Sync/Learn model responds in accordance with the presently relevant weight configurations. Weight configurations created in previous learning events might interfere with the new task, in line with interference theory. At the same time, a novel synchronization needs to be set up (by means of θ-frequency bursts) upon a task switch to accommodate the new task demands, in accordance with the reconfiguration theory.

RT and error rate decrease as a function of practice. This behavioral improvement is consistent with empirical (Crossman, 1959; Logan, 1992; Snoddy, 1926) and computational (Ashby et al., 2007; Verbeke & Verguts, 2019) work. Based on this work, one would expect that RT decreases following a power law and that accuracy increases as a function of practice. Across our three paradigms, we see a clear practice effect for the Sync/Learn models consistent with these empirical trends. For instance, in the extensive learning task we observe an approximate power-law function in the RT as a function of stimulus number (Figure 4A). As mentioned earlier, this decrease in RT is not apparent in the other model results for all paradigms. For example, in the Stroop paradigm, RT fluctuates around a constant value throughout the experiment for the No sync/Learn model (Figures 5A and 5B). We hypothesize that this can be explained by the norm of the weights that remains approximately constant. As a result, the No sync/Learn model has a relatively constant RT, but accuracy varies since this does not depend (only) on the weight norm. Thus, especially the Sync/Learn model is consistent with empirically observed practice effects.

Several authors have related neural synchronization to binding. Distant neurons in the cat visual cortex synchronize in a 40 – 60 Hz frequency band, which allows coordinated neuronal firing (Engel et al., 1991). In macaque monkeys, neurons activated by an attended stimulus showed increased γ-band synchronization (Fries et al., 2001). Increased γ-band synchronization between parieto-occipital and frontotemporal regions was found when humans successfully recognized a human face (Rodriguez et al., 1999), while this synchronization was absent when rotated faces were shown. Synchronization was also present when a motor response was initiated towards a stimulus. Notably, a period of desynchronization occurred between these two synchronization phases suggesting an uncoupling of neural ensembles to enable a shift from one neuronal state (perception) to another (action). Taken together, these results suggest that binding between brain areas is reflected by an increased synchronization in the γ-band. Recall that synchronization in this paper originates from θ-frequency locked bursts to task-relevant input and output units (all oscillating at a specific γ-frequency). By doing so, task-relevant information (e.g. the color of a stimulus) is bound with the action units, ensuring that this information has an increased impact on the model’s response. Thus, synchronizing allows binding to take place in our models, boosting their performance.

Similar to RT, θ power significantly increases upon a task switch. Computationally, this increase in θ is related to the longer RTs observed after a task switch, as this longer RT allows the MFC θ amplitude to build up to higher values. Changes in MFC θ power are often interpreted from a cognitive control perspective. Cavanagh and Frank (2014) note that the need for cognitive control is conveyed by frontal midline θ power (4 – 8 Hz), presumably originating from the MFC. The authors also implicate θ in the exertion of cognitive control. Consistently, stimulus-locked frontal θ power was significantly larger in novel relative to standard stimuli; in high conflict relative to low conflict trials; and in incorrect relative to correct trials (Cavanagh et al., 2012). Other work supports the involvement of θ oscillations in cognitive control. In a cued-trials task-switching paradigm, θ increased when preparing to switch but not when a task was repeated (Cooper et al., 2019). Increased θ power was found in all interference situations across three distinct paradigms (Nigbur et al., 2011). Taken together, there is strong evidence for an association between θ power and cognitive control. Consistent with these data, in our Sync/Learn model, θ power increases when an experimental trial proceeds without response. Indeed, a long time period without a response may indicate that additional control is needed, and in this model, the probability of control is thus increased at such occasions. Future work will have to investigate in humans to what extent an extended non-response is indeed interpreted as a signal to increase cognitive control and thus θ.

So far, the role of θ power in repeated practice remains unclear. A study in rodents (Tort et al., 2009) investigating how rats learned to associate items with their spatial context showed that normalized θ power was significantly lower in the last 30 learning trials, relative to the first 30 learning trials. Kendrick et al. (2011) trained sheep on a visual discrimination task and reported that θ increased after learning. In humans, results are also equivocal. Wascher et al. (2014), investigating mental fatigue in human subjects, reported increasing frontal θ power as a function of time-on-task. The authors suggested that this increased θ reflects an increased effort to perform satisfactorily on the ongoing task. A recent meta-analysis by Tran et al. (2020) suggests that large increases in θ are observed in a majority of paradigms investigating mental fatigue. These changes are seen in frontal, central and posterior regions of the brain. Tran et al. relate the increases in θ to sustained attention. Another recent study (Arnau et al., 2021) indicates that intertrial θ power increases over time, in line with maintaining focus, but stimulus-locked θ actually decreased over time. Hence, a difference was found between time-on-task θ and (non-) task-related θ. While extensive learning and mental fatigue might be correlated, we highlight that the results of these studies might not generalize to the study of extensive learning. Clarke et al. (2018) let human subjects repeatedly learn arbitrary stimulus-response associations via trial-and-error. Here, frontal midline θ was highest during initial learning, and declined as learning took place. Similar results were found in a study where human subjects had to judge a sequence of objects (Crivelli-Decker et al., 2018). Some sequences were consistent (the order of objects was fixed) while others were random. A decrease in θ was observed following decisions on consistent sequences, but not for random sequences. Interestingly, this decrease was only noted in the second half of the trials. Finally, in Huycke et al. (2021) arbitrary stimulus-response associations were learned and repeated both within and between experimental blocks. Here, stimulus-locked θ power decreased over experimental blocks, though not within experimental blocks. We might be able to use our model results to disambiguate how θ relates to extensive learning. We note that in our extensive training model results, θ power decreases with practice (Figure 4E) following a function similar to RT (Figure 4A). Considering θ power as a function of practice on a slower (between blocks) timescale, we see similar dynamics both in the Stroop paradigm (Figures 5E and 5F) and the WCST paradigm (Figures 6E and 6F). In sum, our model captures θ dynamics observed in some experimental paradigms, but not all. Future work will be necessary to determine when θ increases or decreases across time, and at which time scales.

Besides binding, also unbinding processes are key for cognition. To study these, Pastötter et al. (2021) employed a distractor-response binding task and manipulated response repetition, distractor repetition and the prime-probe interval. Their correlational results suggested that post-movement β band (15 – 25 Hz) event-related synchronization (ERS) was associated with disintegration of event files, and thus with the unbinding of event files. In contrast, Rodriguez et al. (1999) suggested a crucial role for the gamma frequency in unbinding. At computational level, Verbeke and Verguts (2019) proposed that unbinding could rely on the same frequency as binding (irrespective of whether that is θ, β, or indeed any other frequency). Taken together, empirical and computational results suggest that oscillations in different frequency bands might be implicated in unbinding of features, but their exact implementation remains to be determined.

In conclusion, we found that learning plays an essential role in simulated behavioral and neural metrics, but the impact of synchronization depends on the task at hand. More precisely, synchronization boosts behavioral performance only in tasks with non-orthogonal inputs, because it diminishes the detrimental impact of catastrophic forgetting on model performance. Our results furthermore suggest that both our behavioral and neural results are modulated by exerted cognitive control and practice effects. Our models provide new insights and hypotheses on how learning and synchronization are modulated by practice and cognitive control.

## Data Accessibility Statement

The data that support the findings of this study are openly available in Zenodo at 10.5281/zenodo.5884290. All scripts used to simulate, process, analyze and visualize model data are stored on GitHub: https://github.com/phuycke/theta_learning_syncing.

## Additional File

The additional file for this article can be found as follows:

10.5334/joc.239.s1 Supplementary materials. Figures s1–s4.
