# Supplementary material for: Computational Investigations of Learning and Synchronization in Cognitive Control
Source: J Cogn. 2022 Sep 30;5(1):44. doi: 10.5334/joc.239 (PMC9524294; doi:10.5334/joc.239)
Supplement: Supplementary materials. — Figures s1–s4. [file joc-5-1-239-s1.pdf]

Supplementary materials

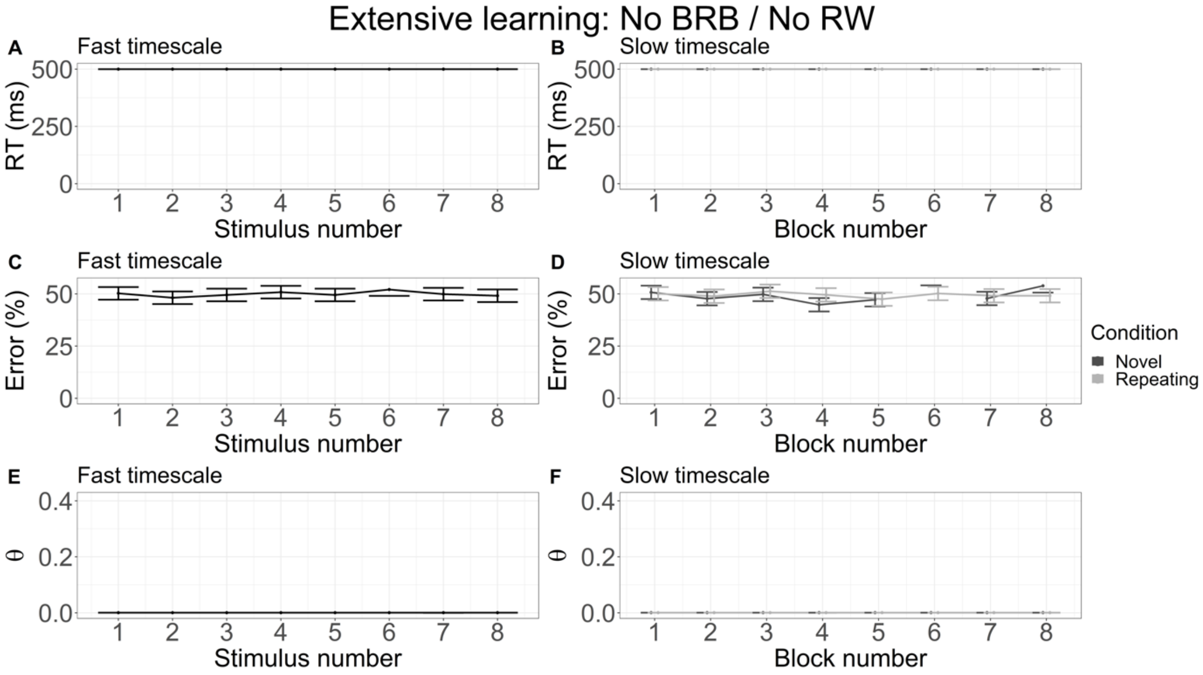

Figure S1. Behavioral and neural simulation results for No Sync / No Learn model performing the extensive learning paradigm.

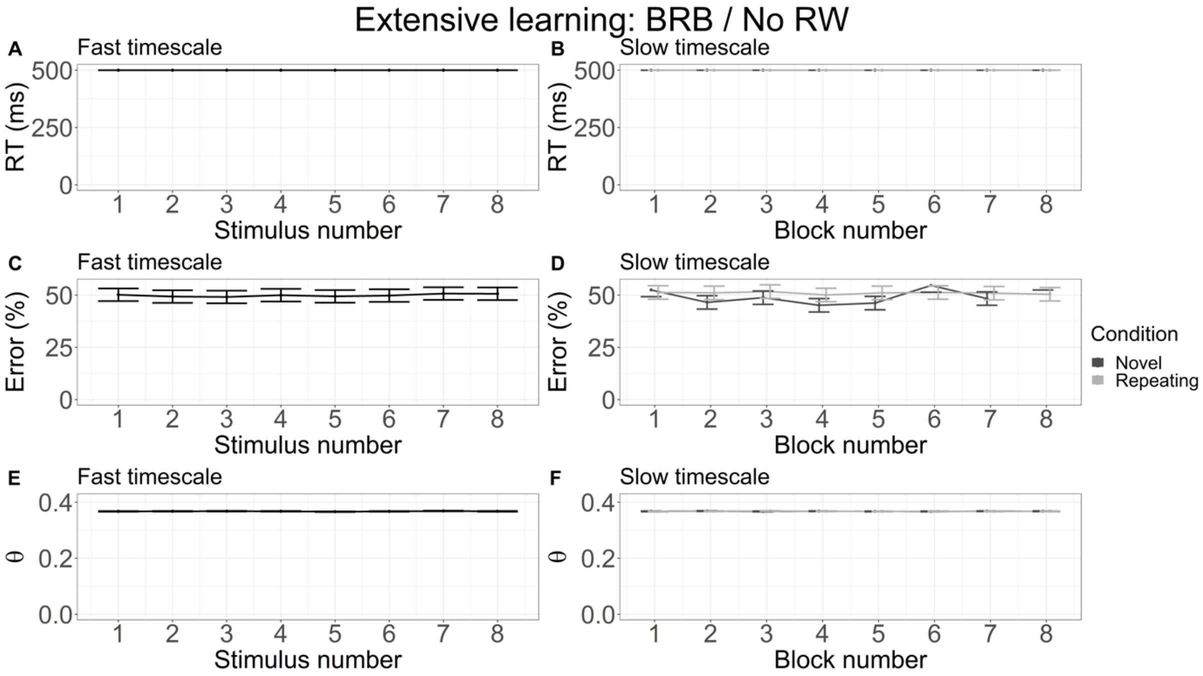

Figure S2. Behavioral and neural simulation results for Sync / No Learn model performing the extensive learning paradigm.

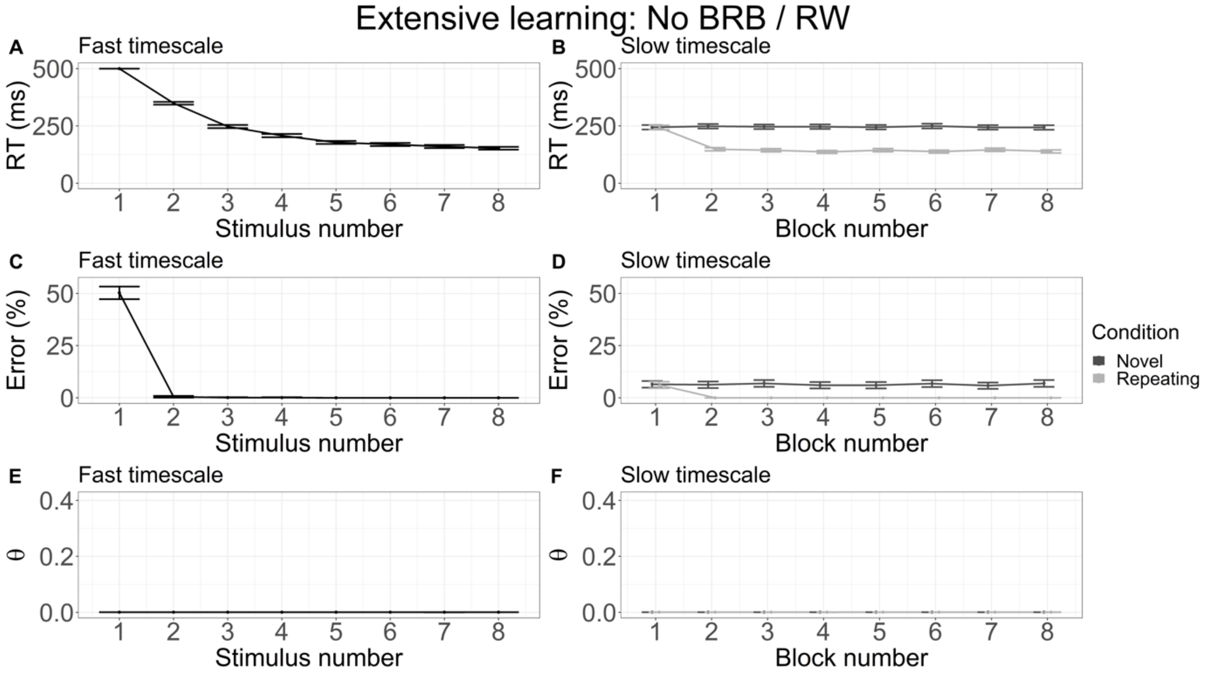

Figure S3. Behavioral and neural simulation results for No Sync / Learn model performing the extensive learning paradigm.

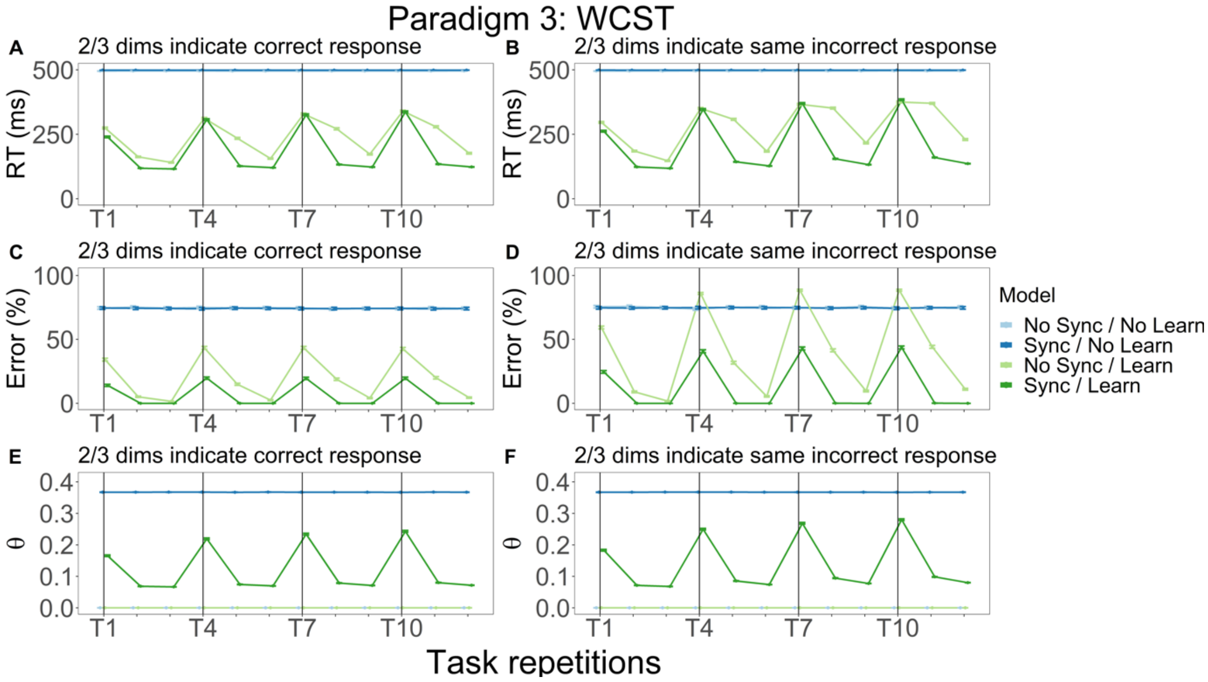

Figure S4. Behavioral and neural simulation results for the other incongruency levels in the WCST paradigm. In the left column, data is shown for trials where two out of three stimulus dimensions point to the correct response. The right column displays data for trials where two out of three stimulus dimensions point to the same incorrect response.
